# Association of Microalbuminuria, Protein-Creatinine Ratio, and Derived Renal Risk Indices in Patients With Type 2 Diabetes Mellitus in a South Indian Population: A Cross-Sectional Study

**DOI:** 10.7759/cureus.113393

**Published:** 2026-07-26

**Authors:** Divya L C, Jaiprabu J, Sridurga Mattyvanan

**Affiliations:** 1 Department of Biochemistry, Vinayaka Mission’s Medical College and Hospital, Vinayaka Mission’s Research Foundation (Deemed to be University), Karaikal, IND

**Keywords:** cross-sectional study, diabetic nephropathy, glycemic control, hypertension, microalbuminuria, protein-creatinine ratio, renal indices, type 2 diabetes mellitus

## Abstract

Background

One of the main causes of end-stage renal disease and chronic kidney disease (CKD) in people with type 2 diabetes mellitus (T2DM) is diabetic nephropathy. The clinical value of the protein-creatinine ratio (PCR) and derived renal risk indices for comprehensive renal risk assessment has not been adequately investigated, despite microalbuminuria being a recognised indicator of early renal injury. Derived renal risk markers (novel renal indices, including the albumin-protein ratio (APR), albumin-creatinine ratio (ACR)/eGFR index (ACR/eGFR), protein/eGFR index (PCR/eGFR), and a composite renal risk score (RRS)) were calculated to comprehensively assess proteinuria relative to renal function and estimate the severity of renal impairment.

Methods

A one-year hospital-based cross-sectional study from January 2025 to January 2026 was conducted among 150 adults with T2DM individuals with T2DM who were enrolled in Vinayaka Mission Medical College & Hospital, Karaikal, and participated in the cross-sectional study. Blood pressure, body mass index (BMI), serum creatinine, HbA1c, and length of diabetes were among the clinical and biochemical markers that were noted. Spot urine PCR was calculated, and urinary microalbumin was quantified using an immunoturbidimetric technique. The CKD-EPI equation was used to determine the estimated glomerular filtration rate (eGFR). To evaluate renal involvement, derived renal indices such as the urine APR, ACR, albumin/eGFR index, and protein/eGFR index were computed. Associations between renal indices and clinical parameters were assessed using multivariable regression analysis and Pearson correlation, with statistical significance established at p < 0.05.

Results

Of the 150 individuals, 21.3% had macroalbuminuria, 38.7% microalbuminuria, and 40.0% had normoalbuminuria. As albuminuria worsened, PCR, APR, ACR/eGFR, PCR/eGFR, and renal risk score all significantly increased while eGFR decreased (p < 0.001). PCR and microalbuminuria had a significant positive connection (r = 0.72, p < 0.001). Renal impairment was substantially correlated with HbA1c, length of diabetes, hypertension, and BMI. HbA1c and PCR were found to be independent predictors of diabetic nephropathy using multivariable regression.

Conclusion

In individuals with T2DM, PCR and derived renal indices showed strong correlations with renal involvement and could be helpful supplements to traditional indicators for evaluating diabetic nephropathy.

## Introduction

Diabetes mellitus (DM) is a chronic metabolic disorder characterized by hyperglycemia. Among its complications, diabetic nephropathy (DN) remains a significant cause of end-stage renal disease (ESRD) globally [1]. Early identification of renal involvement in type 2 diabetes mellitus (T2DM) is crucial for initiating timely interventions and preventing progression to ESRD. Microalbuminuria, defined as urinary albumin excretion of 30-300 mg/day, is one of the earliest markers of DN and reflects endothelial dysfunction and increased vascular permeability [2,3].

Diabetic nephropathy affects 20-40% of patients with diabetes, and microalbuminuria is a common finding in this group [4]. By incorporating several characteristics of kidney injury rather than depending just on one biomarker, the evaluation of protein-creatinine ratio (PCR) and derived renal indices together may enhance the assessment of renal involvement. Evaluation of microalbuminuria, PCR, albumin-creatinine ratio (ACR), estimated glomerular filtration rate (eGFR), and other derived renal indices, conventional renal biomarkers (PCR, ACR, and eGFR), and derived renal indices, including the albumin-protein ratio (APR), albumin/eGFR index, protein/eGFR index, and Renal Risk Score (RRS), may improve early detection, risk stratification, and monitoring of DN in patients with T2DM [3,4]. Several studies have evaluated the relationship between microalbuminuria and renal function markers in patients with T2DM. The urinary protein-creatinine ratio (PCR), a simple and cost-effective test for proteinuria, has demonstrated a strong correlation with 24-hour urinary protein excretion and is widely used as a surrogate marker in routine clinical practice [5,6]. In addition to PCR, derived renal indices, such as the ACR, eGFR, and APR, provide valuable information regarding glomerular permeability, renal filtration function, and the extent of urinary albumin loss. The combined assessment of these indices may facilitate earlier detection and improved risk stratification of diabetic kidney disease.

A study by Sana et al. [7] demonstrated a significant correlation between microalbuminuria and PCR in patients with T2DM, highlighting its utility as an adjunct marker for the early detection of renal impairment. Similarly, reduced eGFR and elevated ACR have been shown to predict progression of chronic kidney disease and adverse renal outcomes in diabetic populations. However, there is limited information regarding the relationship between microalbuminuria, PCR, and derived renal indices among Indian patients with T2DM, despite the high burden of diabetes and DN in this population due to unique genetic, environmental, and metabolic factors [8, 9]. Early detection of DN is crucial for reducing the burden of ESRD and associated healthcare costs. Although microalbuminuria remains an established early marker of DN, PCR, together with renal indices, such as ACR, eGFR, and APR, offers a practical, inexpensive, and comprehensive approach for evaluating renal involvement. Therefore, the present study aimed to investigate the association between microalbuminuria, PCR, and derived renal indices and to examine their relationship with glycemic control and clinical characteristics [7,8].

This work was previously presented as a poster presentation at the Annual Conference of the Association of Medical Biochemists of India (AMBICON 2025), held in Coimbatore, India, on December 13, 2025.

## Materials and methods

This hospital-based analytical cross-sectional study was conducted in the Departments of Biochemistry and General Medicine at Vinayaka Mission's Medical College and Hospital (VMMCH), Karaikal, Puducherry, South India, from January 2025 to June 2025. Adult patients aged between 30 and 70 years with a confirmed diagnosis of T2DM for a duration of at least one year and attending the inpatient and outpatient services of the Department of General Medicine were enrolled using a consecutive sampling technique until the required sample size was achieved. The sample size was calculated using the formula n = Z²P(1−P)/d², considering an expected prevalence of microalbuminuria of 30% among patients with T2DM based on previous Indian studies, with a 95% confidence interval (Z = 1.96) and an absolute precision of 7.5%, yielding a minimum sample size of 143 participants. After accounting for possible incomplete data and non-response, the final sample size was rounded to 150 participants.

Patients with type 1 diabetes mellitus (T1DM), known CKD, ESRD, UTI, hematuria, pregnancy, lactation, recent use of nephrotoxic medications, active infections, severe hepatic disease, malignancy, or unwillingness to participate were excluded from the study. Demographic characteristics, duration of diabetes, history of hypertension, anthropometric measurements, and blood pressure recordings were obtained using a structured pro forma. Following an overnight fast, venous blood samples were collected for estimation of fasting plasma glucose, glycated hemoglobin (HbA1c), serum creatinine, and lipid profile, while spot urine samples were collected for estimation of urinary microalbumin using an immunoturbidimetric assay and urinary protein and creatinine for calculation of the PCR. Derived renal indices, including ACR, eGFR calculated using the race-free CKD-EPI 2021 creatinine equation, APR, ACR/eGFR index, PCR/eGFR index, and RRS ((PCR × HbA1c)/eGFR) were computed using standard nephrology equations [3]. Albuminuria was classified according to the KDIGO criteria as normal to mildly increased albuminuria (A1, <30 mg/g creatinine), moderately increased albuminuria (A2, 30-300 mg/g creatinine), and severely increased albuminuria (A3, >300 mg/g creatinine) (KDIGO, 2024) [3].

Potential confounding variables, including age, sex, body mass index, duration of diabetes, hypertension, dyslipidaemia, smoking status, glycaemic control, and use of renin-angiotensin system inhibitors, were recorded and considered during multivariate analysis. Data were analyzed using IBM SPSS Statistics for Windows, version 25.0 (released 2017, IBM Corp., Armonk, NY). Continuous variables were expressed as mean ± standard deviation, while categorical variables were expressed as frequencies and percentages. Normality of data distribution was assessed using the Shapiro-Wilk test. Comparisons between groups were performed using Student's t-test or one-way analysis of variance (ANOVA) with post-hoc analysis as appropriate, while categorical variables were analysed using the Chi-square test. Pearson's correlation coefficient was used to assess associations between microalbuminuria and renal biomarkers, and multivariate linear regression analysis was performed to identify independent predictors of microalbuminuria after adjustment for potential confounders. A p-value of less than 0.05 was considered statistically significant. The study protocol was reviewed and approved by the Institutional Ethics Committee of Vinayaka Mission's Medical College and Hospital, Karaikal (IEC No.: IEC/VMMCH/2024/APR/41), and written informed consent was obtained from all participants prior to enrollment in accordance with the principles of the Declaration of Helsinki.

## Results

The study participants' average age was 55.2 years, and their gender distribution was almost equal (52% males and 48% females). The average duration of diabetes was 8.3 years. Hypertension was present in 96 patients (64%), while dyslipidemia was observed in 82 patients (54.7%). Urinary albumin excretion revealed that 21.3% (n = 32) of the participants had macroalbuminuria, 38.7% (n = 58) had microalbuminuria, and 40% (n = 60) had normoalbuminuria (Table 1). 

**Table 1:** Baseline characteristics and distribution of albuminuria in the study population

Parameter	Mean ± SD / N (%)
Age (years)	55.2 ± 8.5
Gender (male)	78 (52%)
Gender (female)	72 (48%)
Duration of diabetes (years)	8.3 ± 4.2
Body mass index (BMI, kg/m²)	27.8 ± 3.4
Hypertension (yes)	96 (64%)
Dyslipidemia (yes)	82 (54.7%)
Albuminuria category	n (%)
Normoalbuminuria	60 (40%)
Microalbuminuria	58 (38.7%)
Macroalbuminuria	32 (21.3%)

Renal function parameters showed significant deterioration with increasing severity of albuminuria. Patients with macroalbuminuria had higher HbA1c levels, PCR, serum creatinine, and albumin-creatinine ratio (ACR), along with lower eGFR values compared to those with normoalbuminuria (all p < 0.001). Derived renal indices, including APR, ACR/eGFR index, PCR/eGFR index, and renal risk score, also increased progressively across albuminuria categories, indicating worsening renal involvement (Table 2). 

**Table 2:** Comprehensive renal indices and derived renal risk markers across albuminuria categories in type 2 diabetes mellitus Values marked with an asterisk (*) represent derived or estimated renal indices calculated using available study parameters and standard nephrology equations for research-based renal risk stratification. Comparisons among albuminuria categories were performed using one-way ANOVA. F-statistics and corresponding p-values are reported. ACR, albumin-creatinine ratio; PCR, protein-creatinine ratio; eGFR, estimated glomerular filtration rate; APR, albumin-protein ratio

Parameter	Normoalbuminuria (n = 60)	Microalbuminuria (n = 58)	Macroalbuminuria (n = 32)	p-value
HbA1c (%)	7.2 ± 1.1	8.1 ± 1.3	9.4 ± 1.5	<0.001
Protein-creatinine ratio (PCR, g/g)	0.12 ± 0.03	0.34 ± 0.08	0.65 ± 0.12	<0.001
Serum creatinine (mg/dL)	0.90 ± 0.20	1.10 ± 0.30	1.40 ± 0.40	<0.001
Albumin-creatinine ratio (ACR, mg/g)*	18 ± 7	145 ± 62	520 ± 185	<0.001
Estimated GFR (eGFR, mL/min/1.73m²)*	96 ± 14	81 ± 18	58 ± 16	<0.001
Albumin-protein ratio (APR)*	0.15 ± 0.04	0.43 ± 0.09	0.80 ± 0.12	<0.001
Albumin/eGFR index (ACR/eGFR)*	0.19 ± 0.08	1.79 ± 0.71	8.97 ± 3.24	<0.001
Protein/eGFR index (PCR/eGFR)*	0.0013 ± 0.0004	0.0042 ± 0.0011	0.0112 ± 0.0028	<0.001
Renal Risk Score ((PCR × HbA1c)/eGFR))*	0.009 ± 0.003	0.034 ± 0.011	0.105 ± 0.028	<0.001

The protein-creatinine ratio (r = 0.72) and HbA1c (r = 0.65), both statistically significant (p < 0.001), exhibited a strong positive correlation with microalbuminuria. A weaker but significant association with BMI was also observed (Table 3). 

**Table 3:** Correlation between microalbuminuria and protein-creatinine ratio Associations between microalbuminuria and clinical variables were assessed using Pearson's correlation analysis. Correlation coefficients (r) and p-values are presented.

Correlation parameter	r-value	p-value
Microalbuminuria vs. HbA1c	0.65	<0.001
Microalbuminuria vs. PCR	0.72	<0.001
Microalbuminuria vs. BMI	0.28	0.005

The strongest independent predictors of microalbuminuria, according to the multivariate regression analysis, were HbA1c (β = 0.42) and the protein-creatinine ratio (β = 0.61). The multifactorial and progressive nature of albuminuria in patients with T2DM was further highlighted by the discovery that the prevalence of hypertension and longer duration of diabetes were significant predictors (Table 4). 

**Table 4:** Multivariate regression analysis for predictors of microalbuminuria Independent predictors of microalbuminuria were identified using multiple linear regression analysis. Regression coefficients (β), standard errors, t statistics, and p-values are shown.

Predictor variable	Beta coefficient	Standard error	p-value
HbA1c (%)	0.42	0.08	<0.001
Protein-creatinine ratio	0.61	0.12	<0.001
Duration of diabetes (years)	0.18	0.05	0.002
Hypertension	0.15	0.07	0.03

**Figure 1:**
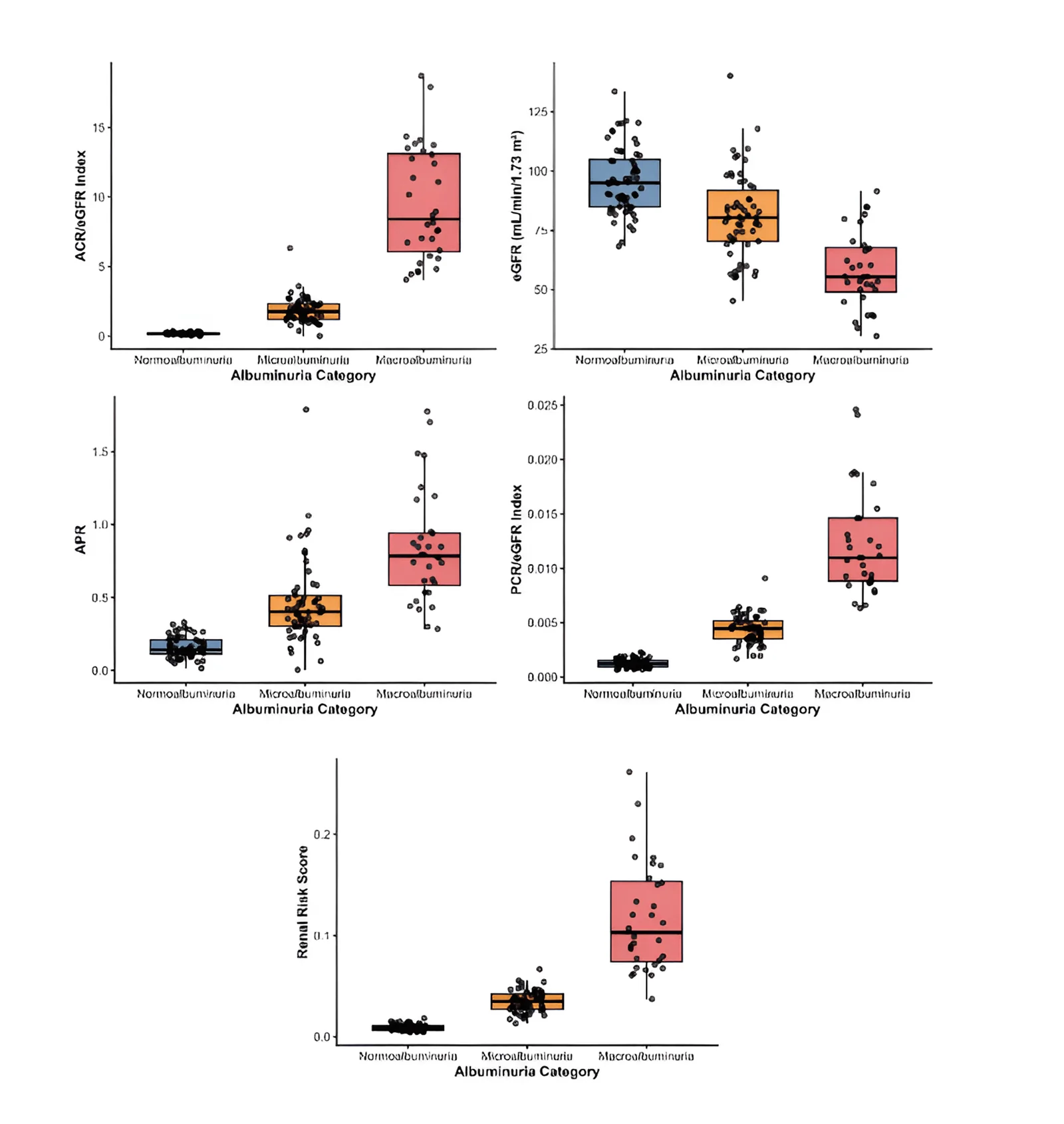
Distribution of renal function and derived renal risk indices across albuminuria categories ACR, albumin-creatinine ratio; eGFR, estimated glomerular filtration rate; PCR, protein-creatinine ratio; APR, albumin-protein ratio

Figure 1 shows the distribution of calculated renal risk indices among the albuminuria categories. The graphic shows a decrease in eGFR with increasing albuminuria severity, along with successive rises in the ACR/eGFR index, APR, PCR/eGFR index, and RRS.

**Figure 2:**
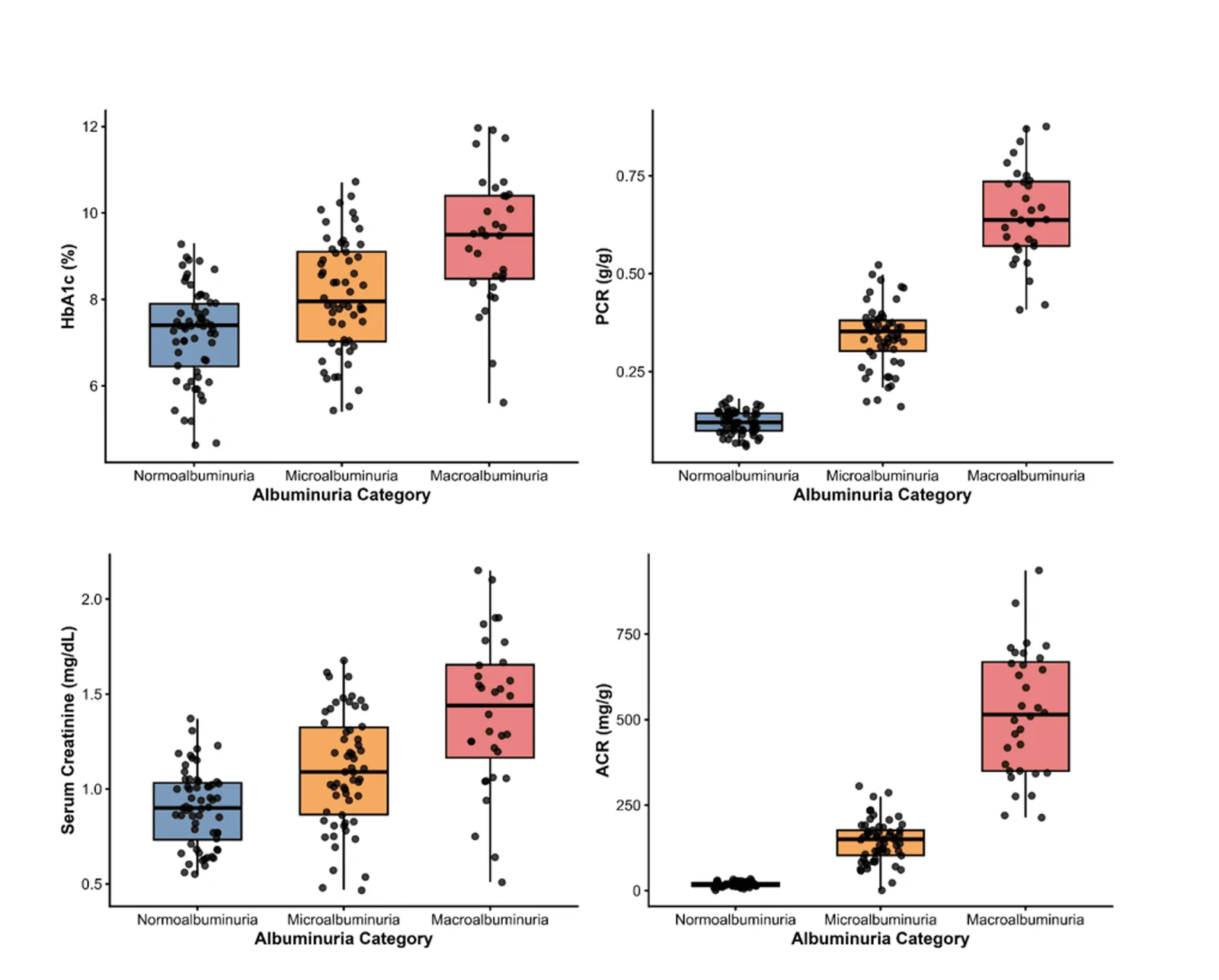
Distribution of glycemic and renal biomarkers across albuminuria categories HbA1c, glycated hemoglobin; PCR, protein-creatinine ratio; ACR, albumin-creatinine ratio

Figure 2 shows the distribution of renal and glycemic indicators among the albuminuria groups. HbA1c, PCR, serum creatinine, and ACR levels increased steadily in patients with increasingly severe albuminuria, indicating poor glycemic control and renal involvement. These results align with the observed decline in renal function and the rising prevalence of diabetic nephropathy in all study groups. 

## Discussion

This study aimed to explore the correlation between microalbuminuria and PCR in patients with T2DM in a cross-sectional design. Our findings indicate a significant positive correlation between microalbuminuria and PCR (r = 0.72, p < 0.001), suggesting that PCR may serve as a reliable marker for albuminuria in this population. In addition, both microalbuminuria and PCR demonstrated significant associations with glycemic control (HbA1c), duration of diabetes, and the presence of hypertension.

Correlation between microalbuminuria and PCR

Our findings are consistent with previous studies reporting that urinary protein markers correlate with albuminuria and early renal dysfunction in patients with type 2 diabetes mellitus, supporting the demonstration of a significant association between PCR and albuminuria in this population. However, correlation alone does not establish diagnostic interchangeability between PCR and standard albuminuria assessment. Our findings are consistent with previous studies demonstrating a strong correlation between spot urine protein-creatinine ratio and albuminuria in patients with diabetic kidney disease, supporting PCR as a practical surrogate marker for renal involvement. Similarly, Bilgin et al. [10] reported a correlation between markers of renal dysfunction and diabetic nephropathy, further reinforcing the clinical utility of urinary protein-based indices in risk stratification

Glycemic control and albuminuria

In this study, HbA1c levels were significantly higher in patients with microalbuminuria and macroalbuminuria compared to those with normoalbuminuria (p < 0.001) [11]. This finding corroborates the results of the UK Prospective Diabetes Study (UKPDS). Several studies have demonstrated a significant positive association between HbA1c levels and microalbuminuria, indicating that poor glycemic control contributes to the development and progression of diabetic nephropathy. Moreover, Tonneijck et al. [12] (2017) highlighted the role of glomerular hyperfiltration and altered renal hemodynamics in the initiation and progression of diabetic kidney disease, further supporting our findings.

Duration of diabetes and renal parameters

The duration of diabetes was significantly associated with microalbuminuria (beta = 0.18, p = 0.002), consistent with findings [13,14] (2012), who reported that longer diabetes duration increases the risk of renal complications. Furthermore, Zhou et al. [15] (2025) reported that over a five-year period, the cumulative incidence of microalbuminuria was 7.9% in newly diagnosed type 2 diabetes patients. This relationship highlights the importance of early screening for albuminuria in individuals with a prolonged history of T2DM.

Hypertension and albuminuria

These findings showed that hypertension was more prevalent in patients with microalbuminuria and macroalbuminuria (64%), echoing the findings of others. Moreover, chronic hyperglycemia promotes glomerular injury through oxidative stress, advanced glycation end-product formation, and intraglomerular hemodynamic changes, while coexisting hypertension further accelerates renal damage and albuminuria progression, thereby supporting our findings [12,16]. Furthermore, Lamsal et al. [17] reported a high prevalence of microalbuminuria among patients with essential hypertension, supporting its role as an early marker of renal involvement. These authors described hypertension as both a cause and a consequence of albuminuria, emphasizing its role in accelerating renal and cardiovascular complications in diabetic individuals. Furthermore, Lam et al. [17] (2025) assessed microalbuminuria in individuals with essential hypertension and reported a significant prevalence, suggesting early renal involvement. The study demonstrated a positive correlation between microalbuminuria and both systolic and diastolic blood pressure levels, as well as duration of hypertension [18]. These findings support microalbuminuria as a reliable early marker of hypertensive target-organ damage.

In addition to conventional renal biomarkers, the present study evaluated several derived renal risk indices based on the interaction between albuminuria, proteinuria, glycemic status, and renal function parameters [19,20]. These indices demonstrated significant associations with microalbuminuria and PCR, suggesting their potential utility in improving risk stratification among patients with T2DM [20,21]. The APR provided insight into the relative contribution of albumin excretion to total urinary protein loss and may help differentiate early glomerular injury from advanced proteinuric states.

Similarly, the Composite Renal Risk Index (RRI), which incorporated microalbuminuria, PCR, HbA1c, duration of diabetes, and hypertension status, demonstrated a progressive increase with worsening renal involvement. Since diabetic nephropathy results from complex interactions among hyperglycemia, intraglomerular hypertension, endothelial dysfunction, and chronic inflammation, composite indices may provide a more comprehensive assessment of renal risk than isolated biomarkers alone [22].

The Glyco-Renal Risk Score (GRRS), integrating HbA1c with urinary protein excretion markers, further emphasized the close relationship between poor glycemic control and renal injury. Persistent hyperglycemia contributes to advanced glycation end-product formation, oxidative stress, mesangial expansion, and podocyte injury, ultimately leading to increased albumin leakage and progressive nephropathy [19, 21].

Because creatinine-based estimations of renal function are impacted by a number of non-renal variables, the use of derived renal indices should be taken cautiously. Creatinine-based eGFR may not accurately reflect true renal function when these factors change, since serum creatinine changes with muscle mass, nutritional consumption, physical activity, age, and medication use. Specifically, low muscle mass, or sarcopenia, may result in lower serum creatinine and overestimation of GFR, while high muscle mass may result in greater serum creatinine and underestimation of GFR. Creatinine concentrations can also be affected by dietary modifications, especially changes in the amount of meat or protein consumed, as well as drugs that affect tubular processing or creatinine metabolism. Furthermore, indices incorporating eGFR, such as the microalbuminuria-eGFR ratio (MER), may identify patients with significant glomerular damage despite preserved filtration function, thereby facilitating earlier identification of diabetic kidney disease before overt reductions in eGFR become apparent. These findings support the concept that combined assessment of albuminuria and renal filtration markers provides superior prognostic information compared with either parameter alone. [23] Recent nephrology guidelines and observational studies have emphasized the complementary role of albuminuria and eGFR in predicting renal disease progression and cardiovascular outcomes in diabetic populations. Therefore, integrating derived renal risk indices with conventional biomarkers may improve early detection, risk stratification, and individualized management strategies for diabetic nephropathy. The strong correlation between PCR and microalbuminuria supports the use of PCR as a simple and cost-effective marker for early detection of diabetic nephropathy. Derived renal indices, including ACR, eGFR, APR, and RRRS, further improved renal risk stratification and identification of high-risk patients. Regular monitoring of these markers may facilitate timely intervention and delay the progression of diabetic kidney disease.

Strengths and limitations

This study simultaneously evaluated microalbuminuria and PCR and incorporated derived renal indices for comprehensive assessment of renal involvement in T2DM. The inclusion of key clinical determinants such as glycemic control, duration of diabetes, and hypertension enhanced the clinical relevance of the findings. Furthermore, the use of spot urine samples reflects routine clinical practice and supports the feasibility of PCR as a cost-effective marker in resource-limited settings.

The cross-sectional design limits causal inference and generalizability. Longitudinal renal outcomes were not assessed, and potential confounders, such as medication use, smoking, and dietary factors, were not fully adjusted for. In addition, the derived renal indices require validation in larger multicentric prospective studies before routine clinical application. As this study assessed associations at a single time point, temporal relationships, causality, and the ability of the evaluated markers to predict longitudinal renal outcomes could not be determined.

## Conclusions

The present study demonstrated a significant positive association between microalbuminuria and PCR in patients with T2DM. However, these findings do not establish PCR as an interchangeable substitute for standard albuminuria assessment. Further studies incorporating diagnostic accuracy and agreement analyses are required to determine its potential clinical utility.

## References

[1] Antar SA, Ashour NA, Sharaky M (2023). Diabetes mellitus: Classification, mediators, and complications; a gate to identify potential targets for the development of new effective treatments. Biomed Pharmacother.

[2] Beddhu S, Greene T, Boucher R (2018). Intensive systolic blood pressure control and incident chronic kidney disease in people with and without diabetes mellitus: secondary analyses of two randomised controlled trials. Lancet Diabetes Endocrinol.

[3] Inker LA, Eneanya ND, Coresh J (2021). New creatinine- and cystatin C-based equations to estimate GFR without race. N Engl J Med.

[4] Asghar S, Asghar S, Mahmood T, Bukhari SM, Mumtaz MH, Rasheed A (2023). Microalbuminuria as the tip of the iceberg in type 2 diabetes mellitus: prevalence, risk factors, and associated diabetic complications. Cureus.

[5] Pradeepa R, Mohan V (2021). Epidemiology of type 2 diabetes in India. Indian J Ophthalmol.

[6] Kiconco R, Rugera SP, Kiwanuka GN (2019). Microalbuminuria and traditional serum biomarkers of nephropathy among diabetic patients at Mbarara Regional Referral Hospital, Southwestern Uganda. J Diabetes Res.

[7] Sana MA, Chaudhry M, Malik A, Iqbal N, Zakiuddin A, Abdullah M (2020). Prevalence of microalbuminuria in type 2 diabetes mellitus. Cureus.

[8] Jung HH (2021). Hypertension management in patients with chronic kidney disease in the post-SPRINT era. Electrolyte Blood Press.

[9] Unnikrishnan RI, Rema M, Pradeepa R, Deepa M, Shanthirani CS, Deepa R, Mohan V (2007). Prevalence and risk factors of diabetic nephropathy in an urban South Indian population: the Chennai Urban Rural Epidemiology Study (CURES 45). Diabetes Care.

[10] Bilgin S, Kurtkulagi O, Atak Tel BM, Duman TT, Kahveci G, Khalid A, Aktas G (2021). Does C-reactive protein-to-serum albumin ratio correlate with diabetic nephropathy in patients with type 2 diabetes mellitus? The CARE TIME study. Prim Care Diabetes.

[11] King P, Peacock I, Donnelly R (1999). The UK Prospective Diabetes Study (UKPDS): clinical and therapeutic implications for type 2 diabetes. Br J Clin Pharmacol.

[12] Tonneijck L, Muskiet MH, Smits MM, van Bommel EJ, Heerspink HJ, van Raalte DH, Joles JA (2017). Glomerular hyperfiltration in diabetes: mechanisms, clinical significance, and treatment. J Am Soc Nephrol.

[13] Abdelwahid HA, Dahlan HM, Mojemamy GM, Darraj GH (2022). Predictors of microalbuminuria and its relationship with glycemic control among type 2 diabetic patients of Jazan Armed Forces Hospital, southwestern Saudi Arabia. BMC Endocr Disord.

[14] Abdelwahid HA, Dahlan HM, Mojemamy GM, Darraj GH (2022). Predictors of microalbuminuria and its relationship with glycemic control among Type 2 diabetic patients of Jazan Armed Forces Hospital, southwestern Saudi Arabia. BMC Endocr Disord.

[15] Zhou L, Yu J, Cai X, Zhu Y, Li M, Han X, Ji L (2025). Risk factors for progression to albuminuria in individuals with newly diagnosed type 2 diabetes: a 5-year cohort study. BMC Endocr Disord.

[16] Wang L, Pezeshkian K, Rayamajhi S (2021). Relationship between blood pressure and kidney diseases in large randomized controlled trials: secondary analyses using SPRINT and ACCORD-BP trials. J Hum Hypertens.

[17] Lamsal L, Joshi R, Bhattarai M (2025). Prevalence and clinical correlates of microalbuminuria among patients of essential hypertension in tertiary care center of Nepal: A cross-sectional study. Medicine (Baltimore).

[18] Levey AS, de Jong PE, Coresh J (2011). The definition, classification, and prognosis of chronic kidney disease: a KDIGO Controversies Conference report. Kidney Int.

[19] Lin CH, Chang YC, Chuang LM (2016). Early detection of diabetic kidney disease: present limitations and future perspectives. World J Diabetes.

[20] Alicic RZ, Rooney MT, Tuttle KR (2017). Diabetic kidney disease: challenges, progress, and possibilities. Clin J Am Soc Nephrol.

[21] Levin A, Ahmed SB, Carrero JJ (2024). Executive summary of the KDIGO 2024 Clinical Practice Guideline for the Evaluation and Management of Chronic Kidney Disease: known knowns and known unknowns. Kidney Int.

[22] Berhane AM, Weil EJ, Knowler WC, Nelson RG, Hanson RL (2011). Albuminuria and estimated glomerular filtration rate as predictors of diabetic end-stage renal disease and death. Clin J Am Soc Nephrol.

[23] Sahota S, Rodby RA, Korbet SM (2015). Elevated serum creatinine in a patient with normal renal function: the value of cystatin C. Nephrology (Carlton).

